# Supplementary figures and images for: Honey contamination from plant protection products approved for cocoa (Theobroma cacao) cultivation: A systematic review of existing research and methods
Source: PLoS One. 2023 Oct 25;18(10):e0280175. doi: 10.1371/journal.pone.0280175 (PMC10599517; doi:10.1371/journal.pone.0280175)

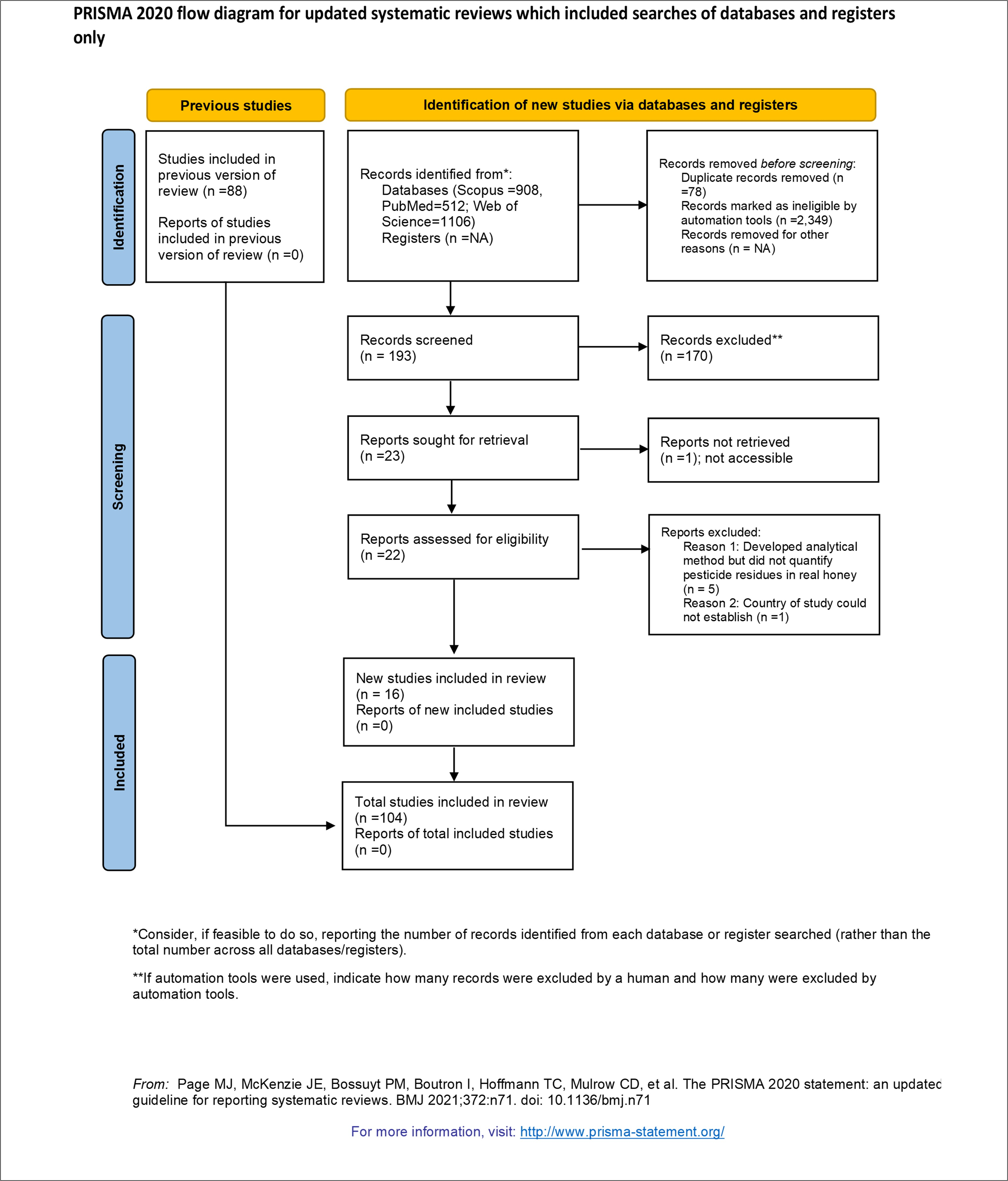

Supplement: S1 Fig — Sixteen 16 publications were retrieved and subsequently added bringing the total papers to 104. (TIF) [file pone.0280175.s002.tif]

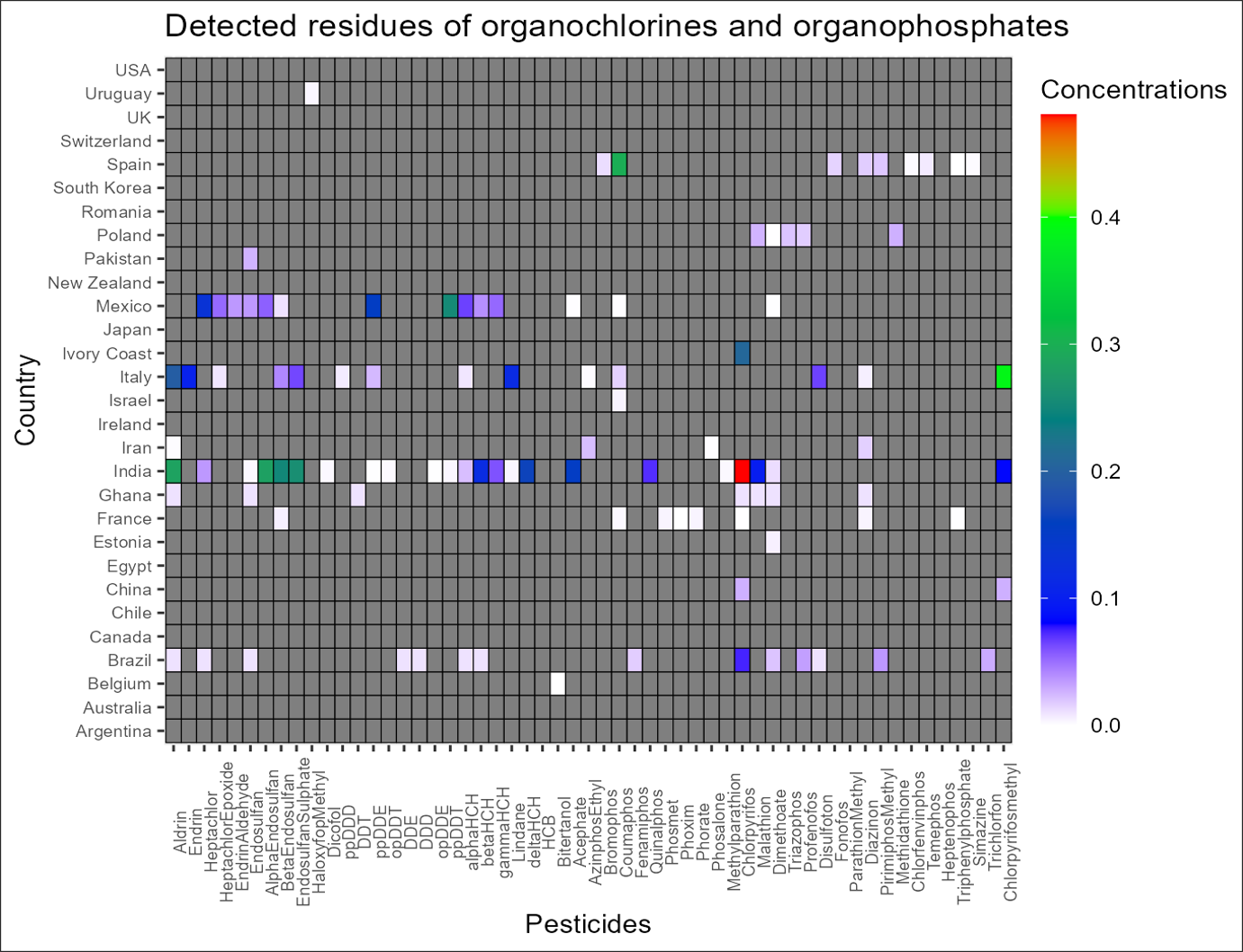

Supplement: S2 Fig — The concentrations of each detected pesticide were averaged per country to generate a single value for each pesticide in each country for the purpose of this visualization. (TIF) [file pone.0280175.s003.tif]

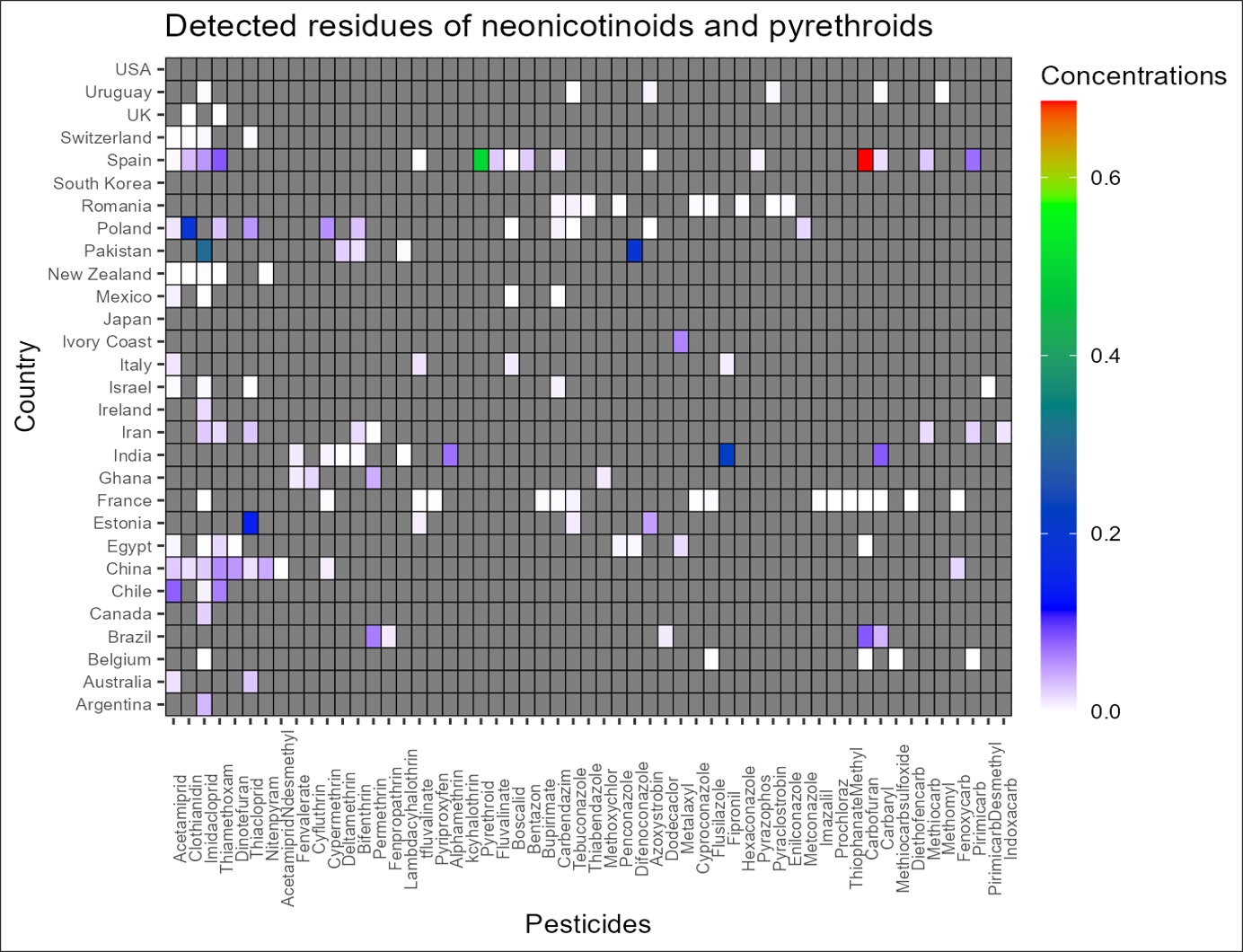

Supplement: S3 Fig — (TIF) [file pone.0280175.s004.tif]

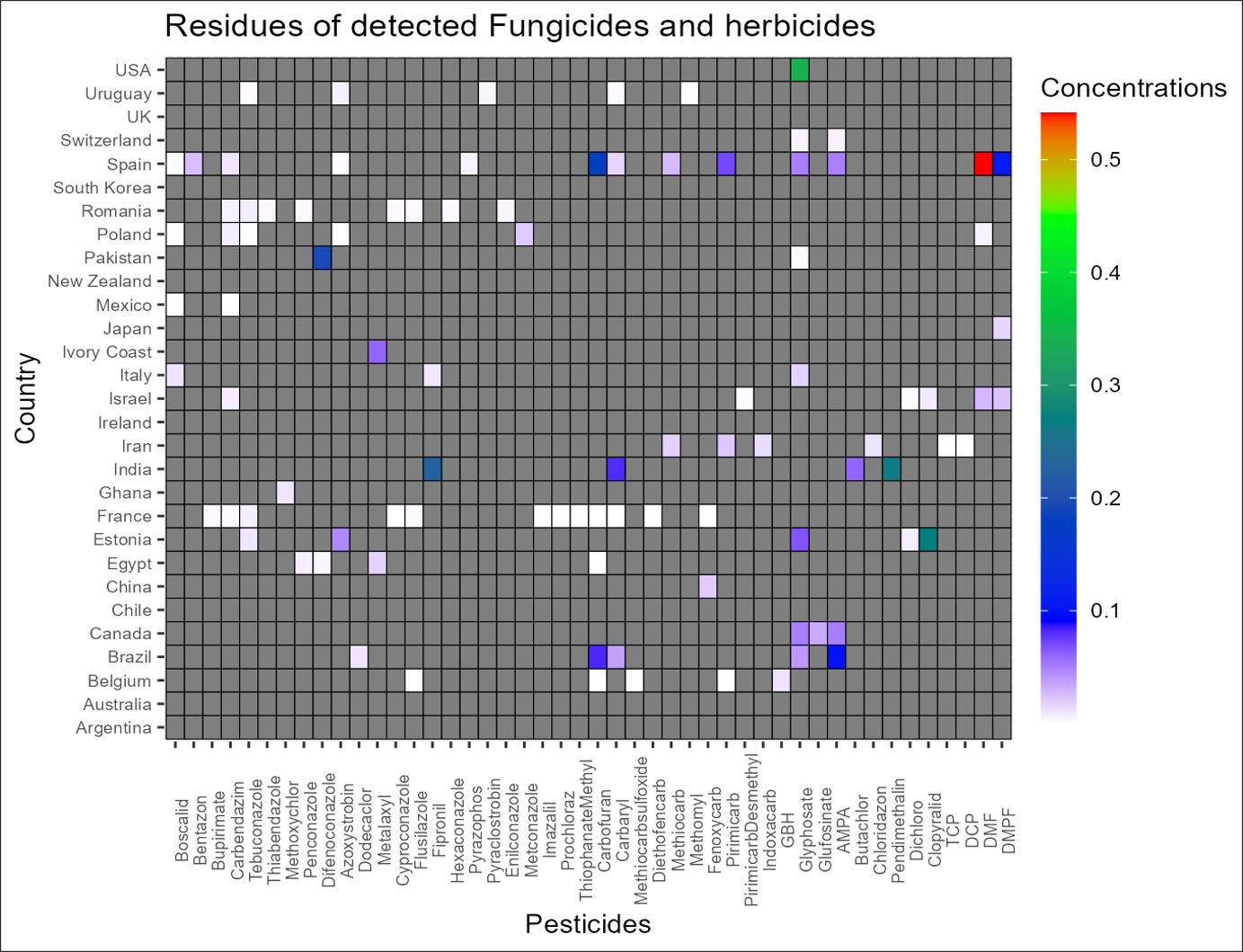

Supplement: S4 Fig — (TIF) [file pone.0280175.s005.tif]

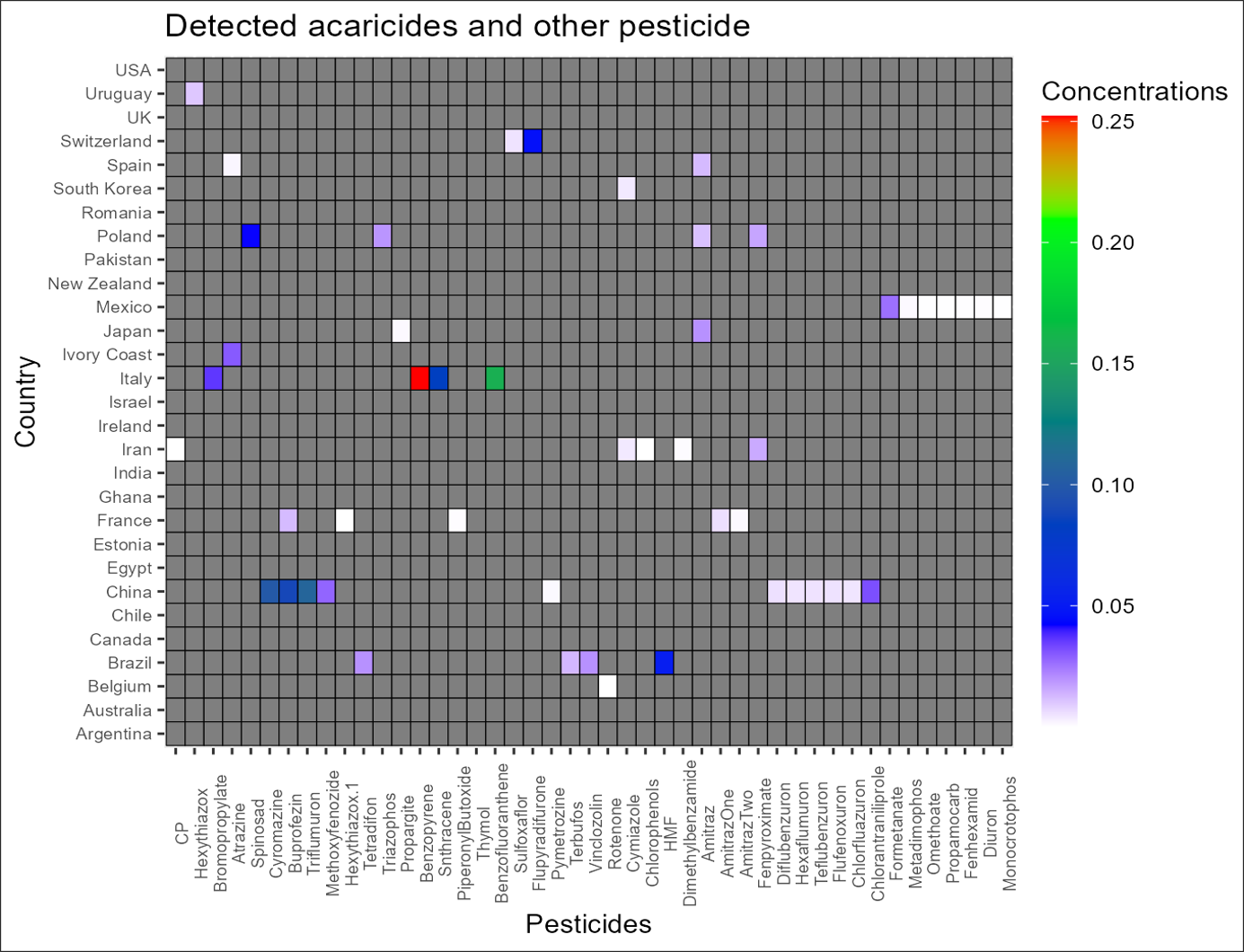

Supplement: S5 Fig — (TIF) [file pone.0280175.s006.tif]
