# Supplementary material for: Honey contamination from plant protection products approved for cocoa (Theobroma cacao) cultivation: A systematic review of existing research and methods
Source: PLoS One. 2023 Oct 25;18(10):e0280175. doi: 10.1371/journal.pone.0280175 (PMC10599517; doi:10.1371/journal.pone.0280175)
Supplement: S1 Table — These search strings were meticulously crafted based on the key pesticides relevant to cocoa cultivation. The selection of these pesticides encompassed twenty-three insecticides, seventeen fungicides, and two herbicides, all of which have been approved for use in cocoa growing. (DOCX) [file pone.0280175.s007.docx]

**S1 Table. Search strings used to retrieve articles from search engines. These search strings were meticulously crafted based on the key pesticides relevant to cocoa cultivation. The selection of these pesticides encompassed twenty-three insecticides, seventeen fungicides, and two herbicides, all of which have been approved for use in cocoa growing**.

| **Search strings** | **Search engine** | **Targeted category** |
| --- | --- | --- |
| (cypermethrin OR capsaicin OR chlorpyriphos OR imidacloprid OR dimethoate OR deltamethrin OR thiamethoxam OR acetaprimid) AND (honey)  (bifenthrin OR pyrethrum OR alpha-cypermethrin OR teflubenzuron OR “lambda cyhalothrin” OR indoxacarb OR chlorantraniliprole OR fipronil OR sulfoxaflor OR etofenprox OR pirimiphosmethyl OR promecarb) AND (honey) | Web of Science Core Collection, PubMed, and Scopus | Insecticides |
| (“copper oxide” OR metalaxyl OR “cuprous hydroxide” OR mancozeb OR maned OR benalaxyl OR benomyl OR “Copper hydroxide” OR Metalaxyl-M OR “copper II hydroxide” OR mefenoxam OR “cupper (I) oxide” OR “dicopper chloride trihydroxide” OR dimethomorph OR fluazinam OR “cuprous hydroxide” OR “cupric hydroxide”) AND (honey) | Web of Science Core Collection, PubMed, and Scopus | Fungicides |
| (glyphosate OR paraquat) AND (honey) | Web of Science Core Collection, PubMed, and Scopus | Herbicides |
