# Supplementary material for: Honey contamination from plant protection products approved for cocoa (Theobroma cacao) cultivation: A systematic review of existing research and methods
Source: PLoS One. 2023 Oct 25;18(10):e0280175. doi: 10.1371/journal.pone.0280175 (PMC10599517; doi:10.1371/journal.pone.0280175)
Supplement: S7 Table — Generally, the concentrations of these pesticides exhibited fluctuations in different studies. It was only in Estonia where azoxystrobin was detected at an identical concentration in two separate different studies. (DOCX) [file pone.0280175.s013.docx]

**S8 Table. An overview of the number and concentrations of pesticide residues detected in countries where multiple studies took place. Generally, the concentrations of these pesticides exhibited fluctuations in different studies. It was only in Estonia where azoxystrobin was detected at an identical concentration in two separate different studies.**

| **Detected Pesticides** | **Concentration mg/kg** | **Country** | **Reference** |
| --- | --- | --- | --- |
| Coumaphos | 0.0051 | Spain | [1] |
| Coumaphos | 0.004 | Spain | [2] |
| Coumaphos | 0.28 | Spain | [3] |
| Coumaphos | 0.036 | Spain | [4] |
| Clothianidin | 0.01 | Spain | [5] |
| Clothianidin | 0.045 | Spain | [6] |
| Imidacloprid | 0.05 | Spain | [5] |
| Imidacloprid | 0.002 | Spain | [2] |
| Thiamethoxam | 0.01 | Spain | [5] |
| Thiamethoxam | 0.144 | Spain | [6] |
| Clothianidin | 0.0013 | Switzerland | [7] |
| Clothianidin | 0.00042 | Switzerland | [8] |
| Imidacloprid | 0.0068 | Switzerland | [8] |
| Imidacloprid | 0.00035 | Switzerland | [7] |
| Thiamethoxam | 0.0025 | Switzerland | [8] |
| Thiamethoxam | 0.00029 | Switzerland | [7] |
| Thiacloprid | 0.014 | Estonia | [9] |
| Thiacloprid | 0.13 | Estonia | [10] |
| Tebuconazole | 0.009 | Estonia | [11] |
| Tebuconazole | 0.005 | Estonia | [9] |
| Azoxystrobin | 0.031 | Estonia | [9] |
| Azoxystrobin | 0.031 | Estonia | [11] |
| Glyphosate | 0.062 | Estonia | [9] |
| Glyphosate | 0.009 | Estonia | [11] |
| 2,4-D | 0.009 | Estonia | [9] |
| 2,4-D | 0.002 | Estonia | [11] |
| Acetamiprid | 0.068 | China | [12] |
| Acetamiprid | 0.0088 | China | [13] |
| Imidacloprid | 0.072 | China | [12] |
| Imidacloprid | 0.0012 | China | [14] |
| Thiacloprid | 0.042 | China | [12] |
| Thiacloprid | 0.0047 | China | [13] |
| Chlorpyrifos | 0.024 | Brazil | [15] |
| Chlorpyrifos | 0.1 | Brazil | [16] |
| Thiamethoxam | 0.202 | Poland | [17] |
| Thiamethoxam | 0.0252 | Poland | [18] |
| Bifenthrin | 0.145 | Poland | [17] |
| Bifenthrin | 0.0152 | Poland | [18] |
| Spinosad | 0.0206 | Poland | [17] |
| Spinosad | 0.02126 | Poland | [18] |
| Imidacloprid | 0.055 | Pakistan | [19] |
| Imidacloprid | 0.736 | Pakistan | [20] |

**References**

1. Gomez-Perez, M.L., et al., *Comprehensive qualitative and quantitative determination of pesticides and veterinary drugs in honey using liquid chromatography-Orbitrap high resolution mass spectrometry.* Journal of Chromatography A, 2012. **1248**: p. 130-138.

2. Juan-Borras, M., E. Domenech, and I. Escriche, *Mixture-risk-assessment of pesticide residues in retail polyfloral honey.* Food Control, 2016. **67**: p. 127-134.

3. Ostiguy, N. and B. Eitzer, *Overwintered brood comb honey: colony exposure to pesticide residues.* Journal of Apicultural Research, 2014. **53**(3): p. 413-421.

4. Lozano, A., et al., *Identification and measurement of veterinary drug residues in beehive products.* Food Chemistry, 2019. **274**: p. 61-70.

5. Sanchez-Hernandez, L., et al., *Residues of neonicotinoids and their metabolites in honey and pollen from sunflower and maize seed dressing crops.* Journal of Chromatography A, 2016. **1428**: p. 220-227.

6. Valverde, S., et al., *Fast determination of neonicotinoid insecticides in beeswax by ultra-high performance liquid chromatography-tandem mass spectrometry using an enhanced matrix removal-lipid sorbent for clean-up.* Microchemical Journal, 2018. **142**: p. 70-77.

7. Mitchell, E.A.D., et al., *A worldwide survey of neonicotinoids in honey.* Science, 2017. **358**(6359): p. 109-+.

8. Kammoun, S., et al., *Ultra-trace level determination of neonicotinoids in honey as a tool for assessing environmental contamination.* Environmental Pollution, 2019. **247**: p. 964-972.

9. Raimets, R., et al., *Pesticide residues in beehive matrices are dependent on collection time and matrix type but independent of proportion of foraged oilseed rape and agricultural land in foraging territory.* Chemosphere, 2020. **238**.

10. Laaniste, A., et al., *Determination of neonicotinoids in Estonian honey by liquid chromatography-electrospray mass spectrometry.* Journal of Environmental Science and Health Part B-Pesticides Food Contaminants and Agricultural Wastes, 2016. **51**(7): p. 455-464.

11. Karise, R., et al., *Are pesticide residues in honey related to oilseed rape treatments?* Chemosphere, 2017. **188**: p. 389-396.

12. Song, S.M., et al., *Simultaneous determination of neonicotinoid insecticides and insect growth regulators residues in honey using LC-MS/MS with anion exchanger-disposable pipette extraction.* Journal of Chromatography A, 2018. **1557**: p. 51-61.

13. Hou, J., et al., *Simultaneous determination of ten neonicotinoid insecticides and two metabolites in honey and Royal-jelly by solid-phase extraction and liquid chromatography-tandem mass spectrometry.* Food Chem, 2019. **270**: p. 204-213.

14. Muhammad, N., et al., *Comprehensive two-dimensional ion chromatography (2D-IC) coupled to a post-column photochemical fluorescence detection system for determination of neonicotinoids (imidacloprid and clothianidin) in food samples.* RSC advances, 2018. **8**(17): p. 9277-9286.

15. Salami, F.H. and M.E.C. Queiroz, *Microextraction in Packed Sorbent for the Determination of Pesticides in Honey Samples by Gas Chromatography Coupled to Mass Spectrometry.* Journal of Chromatographic Science, 2013. **51**(10): p. 899-904.

16. de Pinho, G.P., et al., *Optimization of the liquid–liquid extraction method and low temperature purification (LLE–LTP) for pesticide residue analysis in honey samples by gas chromatography.* Food control, 2010. **21**(10): p. 1307-1311.

17. Barganska, Z., M. Slebioda, and J. Namiesnik, *Pesticide residues levels in honey from apiaries located of Northern Poland.* Food Control, 2013. **31**(1): p. 196-201.

18. Bargańska, Ż., P. Konieczka, and J. Namieśnik, *Comparison of Two Methods for the Determination of Selected Pesticides in Honey and Honeybee Samples.* Molecules, 2018. **23**(10).

19. Farooqi, M.A., et al., *Detection of Insecticide Residues in Honey of Apis dorsata F. from Southern Punjab, Pakistan.* Pakistan Journal of Zoology, 2017. **49**(5): p. 1761-1766.

20. Yaqub, G., et al., *Monitoring and risk assessment due to presence of metals and pesticides residues in honey samples from the major honey producing forest belts and different brands.* Food Science and Technology, 2020. **40**: p. 331-335.
